# Tolerability and age‐dependent toxicokinetics following perinatal hydroxyurea treatment in Sprague Dawley rats

**DOI:** 10.1002/jat.4087

**Published:** 2020-11-25

**Authors:** Madelyn C. Huang, Katie J. Turner, Molly Vallant, Veronica G. Robinson, Yi Lu, Catherine J. Price, Timothy R. Fennell, Melanie A. Silinski, Suramya Waidyanatha, Kristen R. Ryan, Sherry R. Black, Reshan A. Fernando, Barry S. McIntyre

**Affiliations:** ^1^ Division of the National Toxicology Program National Institute of Environmental Health Sciences Durham North Carolina USA; ^2^ RTI International Durham North Carolina USA; ^3^ Social and Scientific Services Durham North Carolina USA

**Keywords:** gestational transfer, half‐life, hydroxyurea, lactational transfer, perinatal, sickle cell disease

## Abstract

Hydroxyurea (HU) is a valuable therapy for individuals with sickle cell anemia. With increased use of HU in children and throughout their lives, it is important to understand the potential effects of HU therapy on their development and fertility. Thus, studies were conducted to identify appropriate doses to examine long‐term effects of prenatal and early postnatal HU exposure and to understand kinetics of HU at various life stages. Pregnant Sprague Dawley dams were administered HU (0–150 mg/kg/day) via oral gavage from gestation days 17 to 21 and during lactation. Pups were dosed with the same dose as their respective dam starting on postnatal day (PND) 10 and up to PND 34. There was minimal maternal toxicity, and no significant effects on littering at any dose of HU. Starting on ~PND 16, offspring displayed skin discoloration and alopecia at doses ≥75 mg/kg/day and lower body weight compared to controls at doses ≥100 mg/kg/day. Gestational transfer of HU was observed, but there was minimal evidence of lactational transfer. Our toxicokinetic studies suggest that the internal dose in offspring may be altered due to age, but not due to sex. The plasma area under the curve, a measure of systemic exposure, at doses tolerated by offspring was threefold to sevenfold lower than the internal therapeutic dose in humans. Therefore, strategies to establish clinically relevant exposures in animal studies are needed. Overall, these data are useful for the design of appropriate nonclinical studies in the future to evaluate the consequences of long‐term HU treatment starting in childhood.

## Abstract 2

Increased use of hydroxyurea (HU) to treat sickle cell disease in children and throughout their lives augments the importance of understanding potential effects of HU on development and fertility. To inform the design of studies to evaluate long‐term safety of HU use in children, tolerability of prenatal and early postnatal HU treatment was evaluated in Sprague Dawley rats. Additionally, studies investigating gestational and lactational transfer of HU and how toxicokinetics of HU vary with age were conducted.

## 1 INTRODUCTION

Hydroxyurea (HU) is currently the most effective therapy for managing sickle cell anemia, a genetic disease that afflicts 100,000 Americans typically of African‐American descent (Ashley‐Koch, Yang, & Olney, 2000). Sickle cell anemia is characterized by the production of a β‐globulin variant, sickle hemoglobulin, which polymerizes when deoxygenated. This polymerization results in sickled erythrocytes that can cause painful vaso‐occlusive crises and significant organ damage due to impaired blood flow. The benefits of HU for sickle cell anemia lie primarily in its ability to increase fetal hemoglobulin production, thus reducing polymerization and vaso‐occlusive events, but through a cytotoxic mechanism. Specifically, HU‐induced erythrocytic cytotoxicity and temporary arrest of hematopoiesis allow for the recruitment of earlier erythroid progenitor cells that still produce fetal hemoglobulin (McGann & Ware, 2015). The use of HU has greatly prolonged the lifespan and increased the quality of life for individuals with sickle cell anemia (Quinn, Rogers, McCavit, & Buchanan, 2010).

In 2017, the FDA approved the use of HU in children 2 years and older with sickle cell anemia based on clinical trials showing efficacy in children (FDA, 2017). However, there are only a few studies that address the safety of long‐term HU use in children. Although the genotoxic and cytotoxic mechanisms of HU are central to its effectiveness for sickle cell anemia, these mechanisms are likely to affect other cells beyond erythrocytes. In particular, HU may affect the proliferation of certain cell populations needed for proper postnatal development of organ systems. Experts have concluded that HU exposure during childhood likely has minimal effects on growth but highlight concerns of impacts on sperm production in individuals who have reached puberty (NTP‐CERHR, 2008). HU may also affect postnatal central nervous system development as it was found to affect neuroblast proliferation and migration, events that are key for cerebellar formation (Marti, Molina, Santa‐Cruz, & Hervas, 2017). Furthermore, it is unknown if there are effects of HU later in life for individuals who start therapy during childhood, such as decreased reproductive ability, impaired neurocognitive capacity, or higher risk of cancer. Investigation of the effect of HU on these types of endpoints would help patients, parents, and providers make more informed decisions on care.

As clinical trials of HU in children are just starting to be conducted, animal studies are valuable for understanding long‐term risks. In addition, toxicokinetic (TK) context is important for study interpretation and for comparison to humans. Although the absorption and distribution of HU have been characterized in animal models, there is limited understanding of the metabolism and kinetics of HU. Moreover, there are limited data evaluating kinetics of HU in children. Metabolism of HU in both animals and humans is thought to occur primarily in the liver, generating urea, carbon dioxide, and nitric oxide (Adamson, Ague, Hess, & Davidson, 1965; Colvin & Bono, 1970; Huang, Yakubu, Kim‐Shapiro, & King, 2006; Jiang et al., 1997). However, the specific enzymes responsible for metabolism have not been identified (Colvin & Bono, 1970; Huang et al., 2006). Thus, it is difficult to predict if there are differences in metabolic capability due to age. Regarding elimination, the half‐life of HU has been extensively studied in adults and ranges from 2 to 5 h in humans (Gwilt & Tracewell, 1998). The kinetics of HU in children are only beginning to be studied (Estepp et al., 2016; Ware et al., 2011). Available TK data of HU in animals do not address the effect of age.

Here, our findings provide data needed to design long‐term safety studies for risk characterization of this important therapy. We conducted a dose‐range finding study focusing on effects associated with prenatal and early postnatal exposure to HU in Sprague Dawley rats. Reproductive toxicity of chemical agents is often evaluated in rats, due to their large litter sizes and high fertility rates. Prenatal exposure to HU was included because it is possible that the mother may undergo HU treatment to manage her disease in the third trimester. We also conducted studies to ascertain gestational and lactational transfer of HU and to assess if the toxicokinetics of HU differ by age in rodents. Overall, these data provide essential information for the design and interpretation of future studies evaluating the safety of long‐term HU use starting in childhood.

## 2 MATERIALS AND METHODS

### 2.1 Chemical procurement and formulation

HU (CAS#127‐07‐1, lot ZU0261) was obtained from Spectrum Chemical Manufacturing Corporation. The identity of the procured material was confirmed using infrared and nuclear magnetic resonance spectroscopy and high‐resolution mass spectrometry. Purity of the chemical was determined to be 99% using high performance liquid chromatography with ultraviolet detection at 200 nm.

The chemical was formulated in distilled water and analyzed using a validated high‐performance liquid chromatography method with UV detection (1–100 mg/ml; *r* > 0.99; relative standard deviation ≤ 10%; relative error ± ≤ 10%) prior to administration to animals. All dose formulations were within 10% of the target concentration and stored at room temperature during conduct of studies. HU was stable in formulations for up to 42 days when stored at room temperature.

### 2.2 Animals

The dose‐range finding study and all TK studies were conducted at RTI International (Research Triangle Park, NC). Animals were housed in facilities fully accredited by the Association for Assessment and Accreditation of Laboratory Animal Care International. Animal procedures were in accordance with the “Guide for the Care and Use of Laboratory Animals” (National Research Council, 2011). Animals were treated humanely and with regard for alleviation of suffering. The study was conducted in compliance with the United States Code of Federal Regulations, Title 21, Part 58: Good Laboratory Practice for Nonclinical Laboratory Studies.

Time‐mated female Hsd:Sprague Dawley® SD® (HSD) rats at 10–13 weeks of age were obtained from Harlan (Indianapolis, IN) and housed in solid‐bottom, polycarbonate cages lined with irradiated hardwood‐chip bedding (Sani‐Chips® cage litter, P.J. Murphy Forest Products, Montville, NJ). Dams were singly housed except when dams were with their respective litters during lactation. In TK studies that extended beyond weaning on postnatal day (PND) 28, pups (8/litter) were cohoused from weaning until termination on PND 34. Irradiated NIH‐07 Certified rodent diet (Zeigler Bros., Inc., Gardners, PA) was available ad libitum throughout the study. Tap water (City of Durham) was available ad libitum via an automatic water delivery system or in bottles. Animal rooms were maintained between 70°F and 74°F, humidity between 46% and 54%, 10 filtered air changes per hour, and on a 12‐h light/dark cycle per day.

#### 2.2.1 Dose‐range‐finding study

##### Study design

Prior to dosing, 40 time‐mated females were randomly assigned to control and treatment groups using a computerized randomization procedure to produce a homogenous distribution of body weights across groups. Eight females/group were administered HU via gavage (5 ml/kg) twice a day (at least 6 h apart) to account for the short half‐life of HU. A total daily dose of 0, 37.5, 75, 100, or 150 mg/kg/day was administered to dams from gestation day (GD) 17 through lactation day (LD) 28. Doses were selected based on literature available at the time of study design. Dosing was started on GD 17 to avoid periods of major organogenesis and to emulate potential prenatal exposure in humans. Offspring were administered the same dose as the dam via twice‐daily gavage from PND 10 until PND 28.

Body weights, clinical observations, and food consumption of dams were measured throughout the study. Gestation length, number of litters, and sex ratio were recorded on PND 0. Live litter size and pup survival were recorded from PND 0 to 28. Body weights and clinical observations of the offspring were recorded on PND 1, 4, 7, and daily from PND 10 to 28.

Animals were euthanized 1 day after PND 28, the last day of dosing. Dams were euthanized without necropsy on LD 28 via CO_2_ asphyxiation with a secondary method to ensure death. Offspring terminated between PND 13 and PND 28 were euthanized by CO_2_ asphyxiation with a secondary method to ensure death. Pups less than 12 days of age were euthanized via intraperitoneal injection containing sodium pentobarbital. Gross necropsies were conducted on offspring found dead or moribund.

##### Data collection and statistical analyses

In‐life data were collected using the ProvantisTM hosted system (Instem Life Sciences Systems, Ltd., Staffordshire, UK).

Statistical analyses were performed using SAS software version 9.4 (SAS Institute, Cary, NC). Dam body weights were analyzed using Jonckheere's (1954) trend test and Williams' or Dunnett's (pairwise) test depending on detection of a significant trend at the 0.01 level (Dunnett, 1955; Williams, 1971, 1972). Gestational length, feed consumption, litter size, and survival endpoints were analyzed using the nonparametric multiple comparison methods of Shirley (1977) as modified by Williams (1986) and Dunn (1964). Jonckheere's trend test was used to assess the significance of dose‐related trends. For reproductive performance endpoints, statistical analysis was performed by Cochran‐Armitage (trend) and Fisher Exact (pairwise) two‐sided tests (Gart, Chu, & Tarone, 1979). Pup weights were first adjusted for litter size and then analyzed with mixed effects linear models with a random litter effect, and the Dunnett‐Hsu procedure (Hsu, 1992) was used to adjust for multiple comparisons. Adjustment of pup weights by litter size was performed by fitting a linear model to pup weights as a function of dose and live litter size on PND 1. The estimated coefficient of litter size was then used to adjust each pup body weight based on the difference between its litter size and the mean litter size. Dosed groups different from control at *p* ≤ 0.05 were considered statistically significant.

For dam body weights and feed consumption endpoints, extreme values were identified by the outlier test of Dixon and Massey (1957). To identify outliers for pup weights, all observations across dose groups were fit to a linear mixed effects model with a random litter effect, and the residuals were tested by dose group for outliers using Tukey's (1977) outer fences method. All flagged outliers were examined, and implausible values were eliminated from the final analyses. For discrete count endpoints, unusual values were manually identified.

#### 2.2.2 TK studies

##### Study design

TK studies were conducted using a limited design to determine (1) the extent of gestational and lactational transfer of HU following treatment of dams and (2) TK differences of HU at varying ages. As in the dose‐range‐finding study, animals were administered HU via gavage twice daily, at least 6 h apart. For most experiments, samples were collected at 10 timepoints (0, 5, 10, 20, 30, 40, 60, 90, 180, and 360 min) after each dose administration from one animal per timepoint (i.e., *N* = 1 per timepoint, 2 dose administrations, 20 animals used overall) (Table 1). Terminal blood collection (exsanguination) by cardiac puncture was conducted on animals anesthetized by CO_2_ inhalation. Blood was collected into tubes containing K_2_EDTA stored on ice; plasma was prepared by centrifugation within 1 h of blood collection and subsequently stored at −80°C until analysis.

**TABLE 1:** Toxicokinetic (TK) study design

Study	Dose, mg/kg^a^	Treatment Period	Samples Collected^b^
Gestational transfer	18.8 and 75	Dams: GD 17	Dam plasma, amniotic fluid (pooled by litter), fetuses (pooled by litter) on GD 17
			*N* = 1/dose/timepoint
Lactational transfer	18.8	Dams: GD 17–LD 14	Dam plasma and pup plasma on LD/PND 14
			Dam: *N* = 1/dose/timepoint
			Pup: *N* = 2/sex/litter/timepoint
TK on PND 21	18.8 and 75	Dams: GD 17–LD 21	Dam plasma and pup plasma on LD/PND 21
		Pups: PND 10–PND 21	
			Dam: *N* = 1/dose/timepoint
			Pup: *N* = 1/sex/litter/timepoint
TK on PND 34	18.8 and 75	Dams: GD 17–LD 28	Pup plasma on PND 34
		Pups: PND 10–PND 34	
			Pup: *N* = 1/sex/litter/timepoint

Abbreviations: GD, gestational day; LD, lactation day; PND, postnatal day.

^a^ Total dose administered. Half the total dose was administered twice daily, at least 6 h apart.

^b^ Samples were collected at 0, 5, 10, 20, 30, 40, 60, 90, 180, and 360 min after each dose administration on the last day of treatment.

To evaluate gestational transfer, dams were administered HU twice (at least 6 h apart) on GD 17 to generate a total dose of either 18.8 or 75 mg/kg HU. Following each dose administration, dam blood was collected via cardiac puncture from one dam per dose group per timepoint. Immediately following blood collection, amniotic fluid (pooled by litter) and fetuses (euthanized by decapitation, pooled by litter) were collected and stored at −80°C.

Lactational transfer was evaluated by dosing dams from GD 17 to LD 14 twice daily to produce a total dose of 18.8 mg/kg/day HU. Pups were not dosed. On LD/PND 14, dam and pup blood were collected via cardiac puncture from one dam and two pups/sex/litter/timepoint.

To evaluate postnatal TK, dams were administered HU twice daily (at least 6 h apart) to produce a total dose of 18.8 or 75 mg/kg/day HU starting on GD 17 until LD 21 or until weaning on LD 28. Pups were dosed with the same dose, also twice daily, as respective dams starting on PND 10 until termination on PND 21 or PND 34. Pups were administered HU starting on PND 10 to parallel the exposure paradigm in the dose‐range finding study. To evaluate TK on PND 21 (juvenile), dam (one/timepoint) and pup (one/sex/litter/timepoint) blood were collected on LD/PND 21; pups and dams were matched by litter for each time point. Dam blood was collected via a nonterminal bleed (from tail vein) for timepoints following the first dose administration on LD 21; after the second daily dose, dam blood was collected via cardiac puncture at each timepoint (i.e., total of 10 dams/dose group). Pup blood was collected via cardiac puncture for all timepoints (one/sex/litter/timepoint). For TK assessment on PND 34 (adolescent), offspring were weaned on PND 28, after which their dams were euthanized without blood collection. On PND 34, pup blood was collected via cardiac puncture (one/sex/litter/timepoint).

##### Quantitation of HU

An analytical method using liquid chromatography‐tandem mass spectrometry (LC‐MS/MS) was qualified to quantitate HU in plasma, amniotic fluid, and fetuses. Solvent and matrix calibration standards were prepared at six concentrations. Triplicate quality control (QC) standards were prepared at two concentrations to assess assay precision and accuracy and six replicate QC standards were prepared at the lowest calibration standard concentration that gave acceptable precision and accuracy, defined as the limit of quantitation (LOQ). The limit of detection (LOD) was calculated as three times the standard deviation of the LOQ.

Fetuses were weighed and homogenized using a tissue grinder until a uniform homogenate was obtained. Stock solution of the internal standard, methoxyurea, was prepared in methanol at 1 mg/ml and diluted in acetonitrile to make a 10‐μg/ml solution. This was further diluted in water (for plasma analysis) or 2% formic acid (for amniotic fluid and fetal homogenate analysis) to make a 50‐ng/ml solution. Two stock solutions of HU were prepared in methanol at 1 and 0.5 mg/ml and diluted in 1:1 acetonitrile:water to prepare two working standards. Six spiking solutions were prepared using alternate working standards. Matrix calibration standards of HU were prepared in duplicate in plasma (40–2,000 ng/ml), amniotic fluid (50–2,000 ng/ml), and fetal homogenate (75–2,000 ng/g) by spiking 50‐μl (50 mg for fetal homogenate) aliquots of sample with 2.5 μl of corresponding HU spiking solution, followed by 100 μl of internal standard solution. For plasma, 1 ml of 1:1 hexane:ethanol was added, and the samples were vortexed and centrifuged for 10 min at 16,000 g at 20°C. The bottom layer was collected, evaporated to dryness, and reconstituted in 50 μl of Mobile Phase A (99.4:0.6 acetonitrile:water, 0.1% aqueous formic acid, and 0.5 mM ammonium formate). For amniotic fluid and fetal homogenate, following addition of the internal standard solution, the samples were vortexed and centrifuged for 10 min at 22,000 g at 4°C. The samples were extracted again using 1 ml of acetonitrile. After another centrifugation, the supernatant was evaporated to dryness and reconstituted in 50 μl of 99.4:0.6 acetonitrile:water, 0.1% aqueous formic acid, and 0.5 mM ammonium formate.

QC samples were prepared in plasma (120 and 1,500 ng/ml), amniotic fluid (150 and 1,500 ng/ml), and fetal homogenate (150 and 1,500 ng/g) similar to that for the matrix standards. Study samples were prepared similar to matrix standards except 2.5 μl of 1:1 acetonitrile:water was added in place of HU spiking solution.

All samples were analyzed using LC‐MS/MS. The instrument used was an Agilent 1100 HPLC coupled to an AB Sciex API‐4000 triple‐quadrupole mass spectrometer. Chromatography was performed on a Waters XBridge Amide column (2.1 × 150 mm, 3.5 μm). Mobile phases A (99.4:0.6 acetonitrile:water, 0.1% aqueous formic acid, 0.5 mM ammonium formate) and B (60:40, water:acetonitrile, 0.1% formic acid, 0.5 mM ammonium formate) were run with a linear gradient from 2% B to 29.7% B in 8 min, ramp to 85% B in 1.5 min, hold for 1 min, return to 2% B in 0.5 min, and hold for 9 min at a flow rate of 0.4 ml/min. The ion source was operated in positive ion mode with a source temperature of 400°C and an ion spray voltage of 5,000 V. Transitions monitored were *m*/*z* 77 → 44 for HU and 91 → 59 for methoxyurea.

Calibration curves relating peak area response ratio of HU to methoxyurea and concentration of HU in matrix (40–2,000 ng/ml; 75–2,000 ng/g) were constructed using a linear regression with 1/X^2^ weighting. The concentration of analyte was calculated using response ratio, the regression equation, initial sample volume, and dilution when applicable. The method was linear for all matrices (*r* ≥ 0.99). The intraday precision measured as % RSD were ≤5.9, ≤8.0, and ≤8.9 and accuracy measured as % RE were ≤ ± 13.1, ≤ ± 6.5, and ≤ ± 13.5 for plasma, amniotic fluid, and fetal homogenate, respectively. Extraction recoveries (%) were 51–59 for plasma, 96–120 for amniotic fluid, and 72–86 for fetuses. Method LOD for plasma and amniotic fluid was 9.2 and 10.6 ng/ml, respectively, and for fetus was 18.4 ng/g. These data support that the method was suitable to quantitate HU in plasma, amniotic fluid, and fetuses following exposure to HU.

##### TK modeling

TK data were analyzed by noncompartmental modeling techniques (Model 200, Phoenix WinNonlin, 6.3). All data above LOD were included in the analysis. When there were plasma data from two pups per sex per time point, average concentrations were used. Two doses (9.4 or 37.5 mg/kg, or half the total dose) administered at 0 and 6 h were used for data modeling. The data collected between 0 and 12 h of the first dosing were used for TK modeling, with data points following the second dosing used in the determination of half‐life.

The following TK parameters were estimated: maximum observed concentration and time at which the maximum concentration was observed (C_max_ and T_max_, respectively), half‐life, and area under the concentration versus time curve (AUC). Dose normalized parameters were calculated based on the total dose administered (18.8 or 75 mg/kg HU).

## 3 RESULTS

All study data are available in the NTP Chemical Effects in Biological Systems (CEBS) database: https://doi.org/10.22427/NTP‐DATA‐NTP‐DATA‐002‐02275‐0007‐0000‐5.

### 3.1 Dose‐range‐finding study

#### 3.1.1 Dam body weight, food consumption, and littering parameters

**FIGURE 1:**
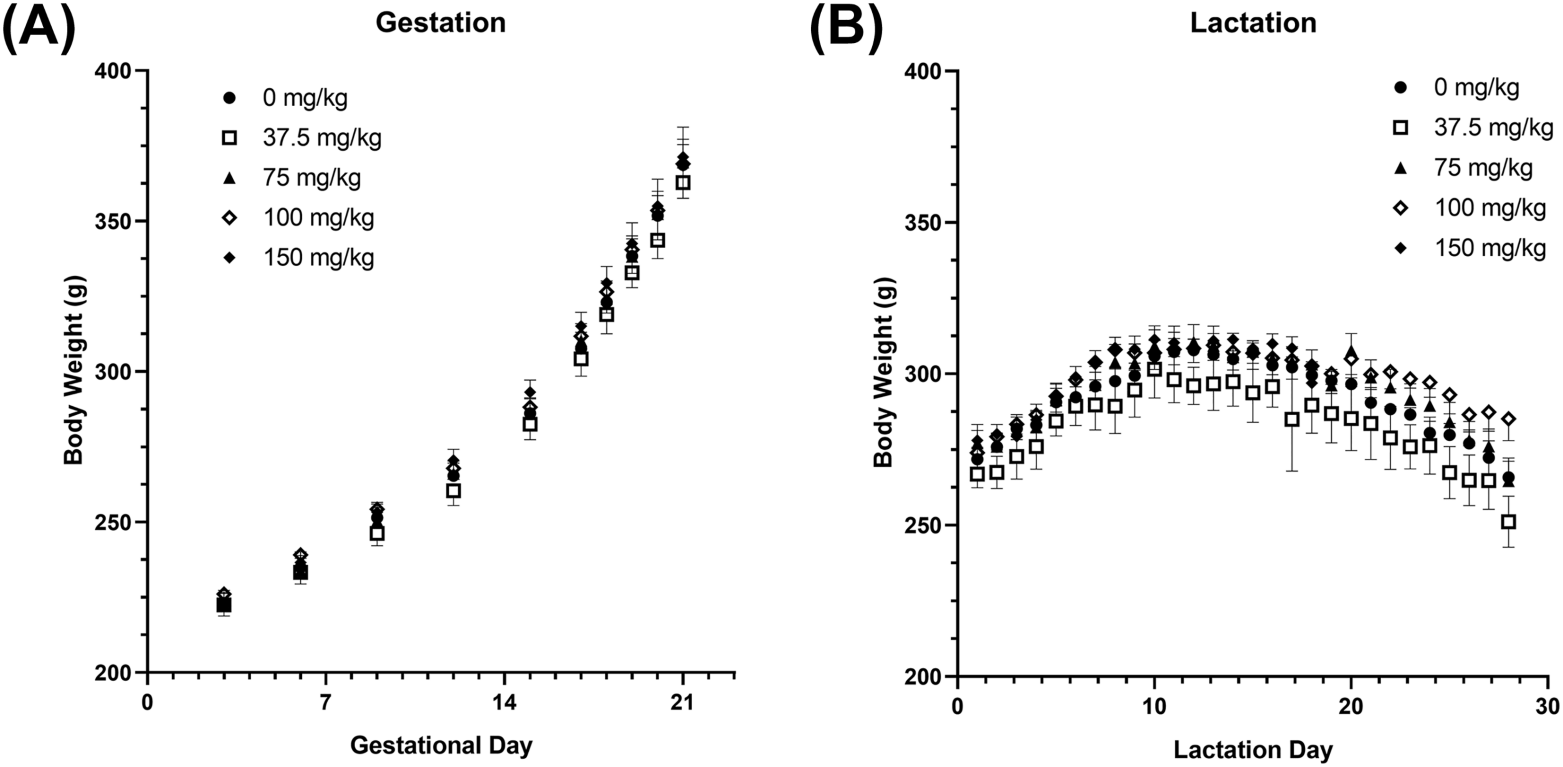
Body weight of dams treated with hydroxyurea during gestation and lactation. Dams (*N* = 6–8) were administered hydroxyurea via oral gavage daily throughout (A) gestation and (B) lactation. Dams in the 150 mg/kg/day group were terminated on lactation days 17 and 18 due to severe decreases in pup body weight. Data shown as mean ± SEM

There were no significant effects of HU on dam survival, body weight, body weight gain, or food consumption during gestation (CEBS Tables I01, I03, I04, I04G, I06). During lactation, there were also no significant effects of HU on maternal survival, body weight (Figure 1), or body weight gain (CEBS I04G). Food consumption in the 100 mg/kg/day group was significantly reduced (20% lower than control) from LD 14 until LD 28, approximately (Table 2). Food consumption was also significantly lower (10% lower than control) in the 75 mg/kg/day group but only during the LD 14–21 period. There was a significant decreasing trend in gestation length with increasing dose and no effect of HU on the number of litters. On PND 0, there was no difference across treatment groups in the number of live pups per litter, sex ratio, or survival ratio (Table 3).

**TABLE 2:** Food consumption (g/kg/day) during lactation of dams administered hydroxyurea

Lactation Day	0 mg/kg/day	37.5 mg/kg/day	75 mg/kg/day	100 mg/kg/day	150 mg/kg/day
*N*	8	5	7	7	6
1–14	174.6 ± 5.3	176.4 ± 4.6	172.4 ± 4.0	163.5 ± 13.9	160.3 ± 15.8
14–21	257.0 ± 7.3^**^	264.7 ± 12.0	231.7 ± 4.4^*^	217.4 ± 20.8^*^	NR
21–28	410.6 ± 22.2^**^	447.3 ± 31.1	352.9 ± 14.4	245.8 ± 27.5^**^	NR
1–28	255.7 ± 9.1^**^	266.8 ± 11.6	233.7 ± 5.5	198.8 ± 18.3^**^	NR

Abbreviation: NR, not reported as group was terminated on PND 17 and 18 due to severe decreases in pup body weight.

* Statistically significant at *p* ≤ 0.05.

** Statistically significant at *p* ≤ 0.01. Significance indicated on control groups indicates a statistically significant trend whereas significance on treatment groups indicates significant pairwise tests compared to the vehicle control group. Statistical analysis performed by Jonckheere (trend) and Shirley or Dunn (pairwise) tests.

**TABLE 3:** Reproductive and litter data after treatment with hydroxyurea starting on gestational day 17

	0 mg/kg/day	37.5 mg/kg/day	75 mg/kg/day	100 mg/kg/day	150 mg/kg/day
Number paired	8	8	8	8	8
Number littered	8	6	8	7	7
Gestation length (% of control)	22.6 ± 0.2^*^ (100%)	22.2 ± 0.2 (98.2%)	22.1 ± 0.0 (97.8%)	22.0 ± 0.2 (97.3%)	22.1 ± 0.1 (97.8%)
Live pups per litter^a^ (% of control)	13.0 ± 1.2 (100%)	12.2 ± 0.8 (98.3%)	13.1 ± 0.5 (99.2%)	12.4 ± 1.9 (114%)	12.1 ± 2.0 (93.3%)
% Male^a^	56.3 ± 7.0	49.7 ± 4.6	52.8 ± 6.1	42.9 ± 8.8	49.1 ± 3.8
Pup survival ratio^a^	0.96 ± 0.04	0.82 ± 0.17	0.80 ± 0.12	0.99 ± 0.01	0.84 ± 0.14

^a^ Evaluated on postnatal day 0.

* *p* ≤ 0.05 significance in the Jonckheere trend test.

#### 3.1.2 Pup clinical observations

Starting on PND 16, alopecia was observed in some offspring at ≥75 mg/kg/day. Alopecia was observed in four litters in both the 75 and 100 mg/kg/day group and ranged from partial to complete hair loss. All litters in the 150 mg/kg/day group had complete hair loss. A yellow discoloration of the skin was also observed in two of seven litters in the 75 mg/kg/day group and in the majority of litters in the 100 and 150 mg/kg/day group starting around PND 18. Distension (swelling) of the abdomen was observed in one pup in the 100 mg/kg/day group and in 17 pups (from two litters) in the 150 mg/kg/day group. Pups in three litters from the top dose group also had increased incidence of bruising on PND 17 and 18 (CEBS Tables I05P).

#### 3.1.3 Pup body weight

**FIGURE 2:**
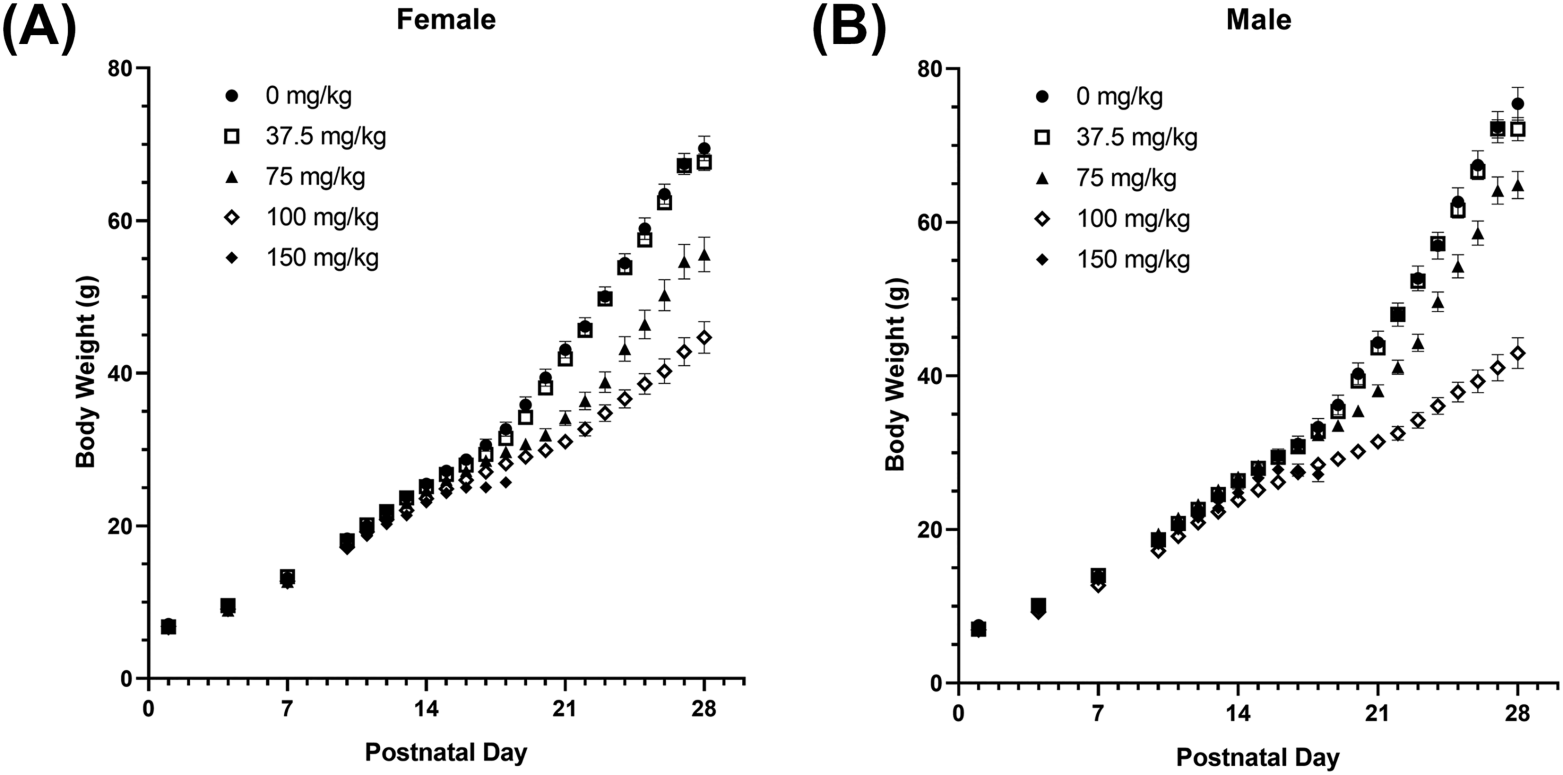
Pup body weight from postnatal day (PND) 1 to PND 28 after exposure to hydroxyurea. Dams were treated with hydroxyurea from gestational day 17 to lactation day 21 and pups were directly dosed with the same hydroxyurea concentration as their mothers from PND 10 to 28. Data shown as mean ± SEM; *N* = 5–8 litters. For both (A) female and (B) male pups, body weight was significantly (*p* ≤ 0.05) lower than control in the 75, 100, and 150 mg/kg/day groups starting on PND 16 and onwards. Pups in the 150 mg/kg/day group were terminated on PND 17 and 18 due to severe decreases in body weight

From PND 1 through 10, there were no HU‐related effects on mean pup weight/litter, by sex and combined. There was a decreasing trend in body weight with increasing dose starting on PND 13 and body weight was significantly lower than control in the 75, 100, and 150 mg/kg/day groups starting on PND 16 (Figure 2). Pups in the 150 mg/kg/day groups were euthanized on PND 17 and 18 due to losses in body weight and adverse clinical observations (i.e., alopecia, skin discoloration, and abdominal swelling). Gross findings in these animals included pale kidney, pale liver, distended stomach, and absence of milk/food.

PND 18 pup body weights were 19%–24% lower in the 100 mg/kg/day group than those in the control group. By PND 21, pup body weight in the 100 mg/kg/day group was significantly lower than control by ~30% and by 38%–44% on PND 28 (Figure 2). Due to the absence of adverse clinical observations, these animals were retained on study. In the 75 mg/kg/day group, body weight was significantly lower starting on PND 18 in males and on PND 19 in females and remained lower until the end of the study. On PND 28, body weight was 20%–22% lower than control. Body weight gain in both sexes was significantly lower in the 75, 100, and 150 mg/kg/day groups starting on PND 10 onwards (CEBS Table R19G).

#### 3.1.4 TK studies

##### Gestational transfer of HU

**FIGURE 3:**
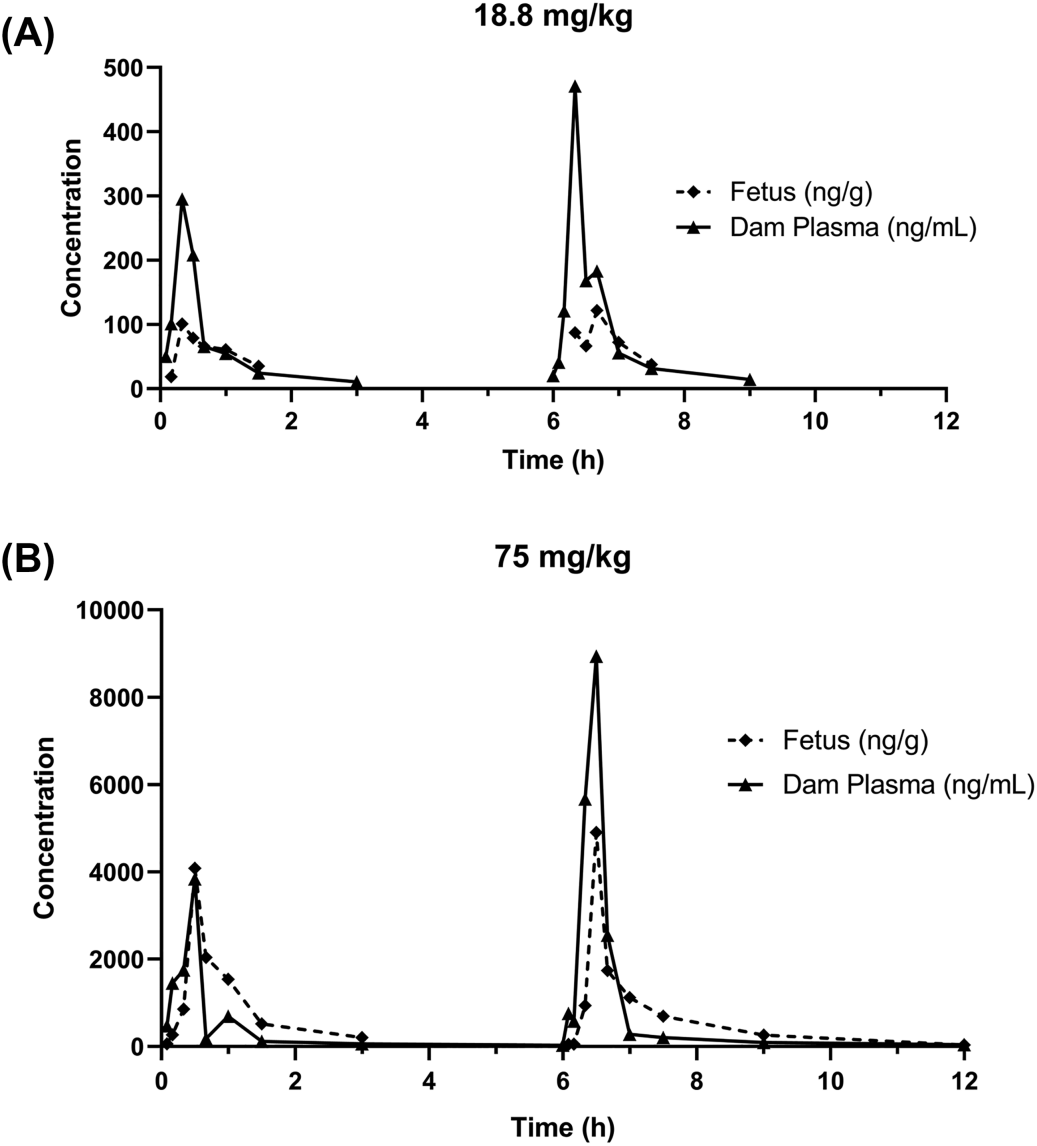
Concentrations of hydroxyurea (HU) in dam plasma and fetuses (*N* = 1/dose/timepoint) on gestational day (GD) 17 measured over time. Dams were administered HU twice a day to equal a total dose of (A) 18.8 mg/kg/day and (B) 75 mg/kg/day from GD 17 to lactation day 21. Half the total dose of HU was administered at time 0 and 6 h. HU in fetuses was below the detection limit of 18.4 ng/g at multiple timepoints in the 18.8 mg/kg dose group

Dams were administered a total of 18.8 or 75 mg/kg HU on GD 17 via oral gavage (two administrations in 1 day), and HU concentrations were measured in dam plasma, amniotic fluid, and fetuses. HU was detected in plasma of dams administered 18.8 mg/kg at all postadministration time points through 3 h; HU concentration was below LOD at other timepoints. In dams administered 75 mg/kg, HU was detected in plasma at all timepoints following each dose administration (Figure 3). Concentrations of HU in the amniotic fluid were below the LOD at all timepoints in the 18.8 mg/kg group. In the 75 mg/kg group, HU concentrations ranged from below LOD to 698 ng/ml (CEBS Table PA48). In fetuses, concentrations of HU were above the LOD starting at 20 min after each dose until 1.5 h postadministration in the 18.8 mg/kg group. In the 75 mg/kg group, HU was detected in fetuses starting at 5 min after dosing until 6 h postadministration (Figure 3).

TK parameters of HU in dam plasma and fetuses are shown in Table 4. Due to poor fitting of available data, meaningful TK parameters were not able to be estimated for amniotic fluid. In dams on GD 17, T_max_ was reached in under 0.5 h in both dose groups. The C_max_ and AUC increased more than proportionally to the dose: There was a 13‐fold and 11‐fold increases in C_max_ and AUC, respectively, despite a ~fourfold increase in dose. The estimated half‐life of HU in pregnant dams was approximately 1 and 2 h, with a longer half‐life in the higher dose group.

**TABLE 4:** Toxicokinetic parameters of hydroxyurea in dam plasma and fetuses or pups on gestational day (GD) 17 and lactation/postnatal day (LD/PND) 14 after oral administration starting on GD 17

Matrix	GD 17				LD/PND 14	
	Dam Plasma		Fetuses		Dam Plasma	Pups
Dose, mg/kg/day^a^	18.8	75	18.8	75	18.8	^b^
T_max_, h	0.33	0.5	0.33	0.5	0.33	^d^
Half‐life, h	1.08	1.84	0.49	1.04	2.29	^d^
C_max_, ng/ml^c^	295	3,840	101	4,080	1,060	^d^
AUC_inf_, ng * h/ml^c^	509	5,689	497	6,321	1918	^d^
Dose‐normalized C_max_, ng/ml/mg/kg^c^ ^,^ ^e^	16	51	5	54	56	^d^
Dose‐normalized AUC_inf_, ng * h/ml/mg/kg^c^ ^,^ ^e^	27	76	26	84	102	^d^

^a^ The half daily target dose was used in modeling, that is, 9.4 and 37.5 mg/kg for 18.8 and 75 mg/kg groups, respectively.

^b^ Pups did not receive HU directly but were from dams administered 18.8 mg/kg/day.

^c^ Fetus units: C_max_, ng/g; AUC_inf_, ng * h/g.

^d^ Parameters not estimated due to values below limit of detection or limit of quantitation (9.2 ng/ml in plasma).

^e^ Dose‐normalized values were calculated by dividing by the total dose administered.

In fetuses on GD 17, the T_max_ of HU occurred before 0.5 h for both dose groups and was similar to that observed in the dams, suggesting rapid transfer from dams to fetuses (Table 4). In addition, the C_max_ and AUC were similar to that in dam plasma in both dose groups demonstrating significant transfer of HU from dams to fetuses. The half‐life of HU in fetuses was also similar to that in dams and ranged from 0.5 to 1.0 h.

##### Lactational transfer of HU

Dams were administered a total of 18.8 mg/kg/day HU via oral gavage (two administrations in 1 day) from GD 17 to LD 14. On LD/PND 14, dam and pup plasma were analyzed at multiple timepoints after each dose administration. In dams, HU concentrations were detectable at all time points through 3 h after dose administration. Although HU was detected in dam plasma on LD 14, HU in pup plasma was below the LOD at all timepoints. TK parameters of HU in dams on LD 14 are shown in Table 4.

##### Postnatal TK evaluations

**FIGURE 4:**
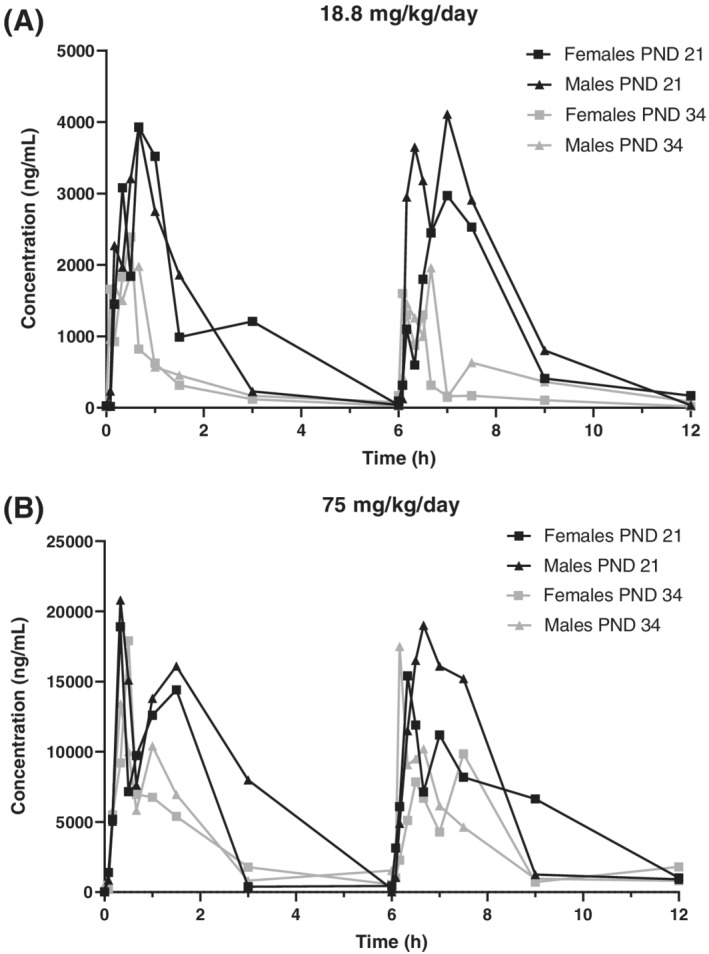
Concentrations of hydroxyurea (HU) in plasma of offspring (*N* = 1/sex/litter/timepoint) on postnatal day (PND) 21 and 34 measured over time. Animals were administered HU twice a day to equal a total dose of (A) 18.8 mg/kg/day and (B) 75 mg/kg/day. Dams were treated from gestational day 17 to lactation day 21; offspring were treated from PND 10 to PND 28. Half the total dose of HU was administered at time 0 and 6 h

Dams were administered a total of 18.8 or 75 mg/kg/day HU via oral gavage (two administrations in 1 day) from GD 17 to LD 21. Pups were administered the same dose as their respective dam starting on PND 10 until termination at either PND 21 or PND 34. HU was detected in dam plasma on LD 21 in the 18.8 mg/kg/day group at all time points through 1 h postadministration and in the 75 mg/kg/day dose group at all timepoints. In PND 21 and PND 34 pups, HU was detected in plasma at all timepoints for both doses following each dose administration (Figure 4). Corresponding TK parameters are shown in Table 5.

**TABLE 5:** Toxicokinetic parameters of hydroxyurea in dam plasma on lactation day (LD) 21 after administration of 18.8 or 75 mg/kg/day starting on gestational day 17 and in pup plasma on postnatal day (PND) 21 and PND 34 after in utero exposure and direct dosing starting on PND 10

Matrix	Dam Plasma		Male pups		Female pups	
Dose, mg/kg/day^a^	18.8	75	18.8	75	18.8	75
PND/LD 21						
T_max_, h	0.17	0.33	0.67	0.33	0.67	0.33
Half‐life, h	0.19	0.78	0.70	1.13	1.22	1.47
C_max_, ng/ml	1,390	4,490	3,930	20,800	3,930	18,900
AUC_inf_, ng * h/ml	1,003	8,680	14,385	86,477	13,641	67,537
Dose‐normalized C_max_, ng/ml/mg/kg^b^	74	60	209	277	209	252
Dose‐normalized AUC_inf_, ng * h/ml/mg/kg^b^	53	116	765	1,153	726	901
PND 34						
T_max_, h	NA	NA	0.67	0.33	0.5	0.5
Half‐life, h	NA	NA	1.53	1.32	1.44	2.28
C_max_, ng/ml	NA	NA	1980	13,400	2,390	17,900
AUC_inf_, ng * h/ml	NA	NA	5,442	41,661	3,340	46,070
Dose‐normalized C_max_, ng/ml/mg/kg^b^	NA	NA	105	179	127	239
Dose‐normalized AUC_inf_, ng * h/ml/mg/kg^b^	NA	NA	289	555	178	614

^a^ The half daily target dose was used in modeling, that is, 9.4 and 37.5 mg/kg for 18.8 and 75 mg/kg groups, respectively.

^b^ Dose‐normalized values were calculated by dividing by the total dose administered.

NA, not applicable.

In dams on LD 21, T_max_ was 0.17 and 0.33 h for the 18.8 and 75 mg/kg/day dose groups, respectively. C_max_ and AUC did not increase in a dose‐proportional manner; there was a slight decrease in the dose‐normalized C_max_ and only a twofold increase in dose‐normalized AUC with increasing dose. The half‐life of HU in dams on LD 21 was under an hour for both dose groups.

**FIGURE 5:**
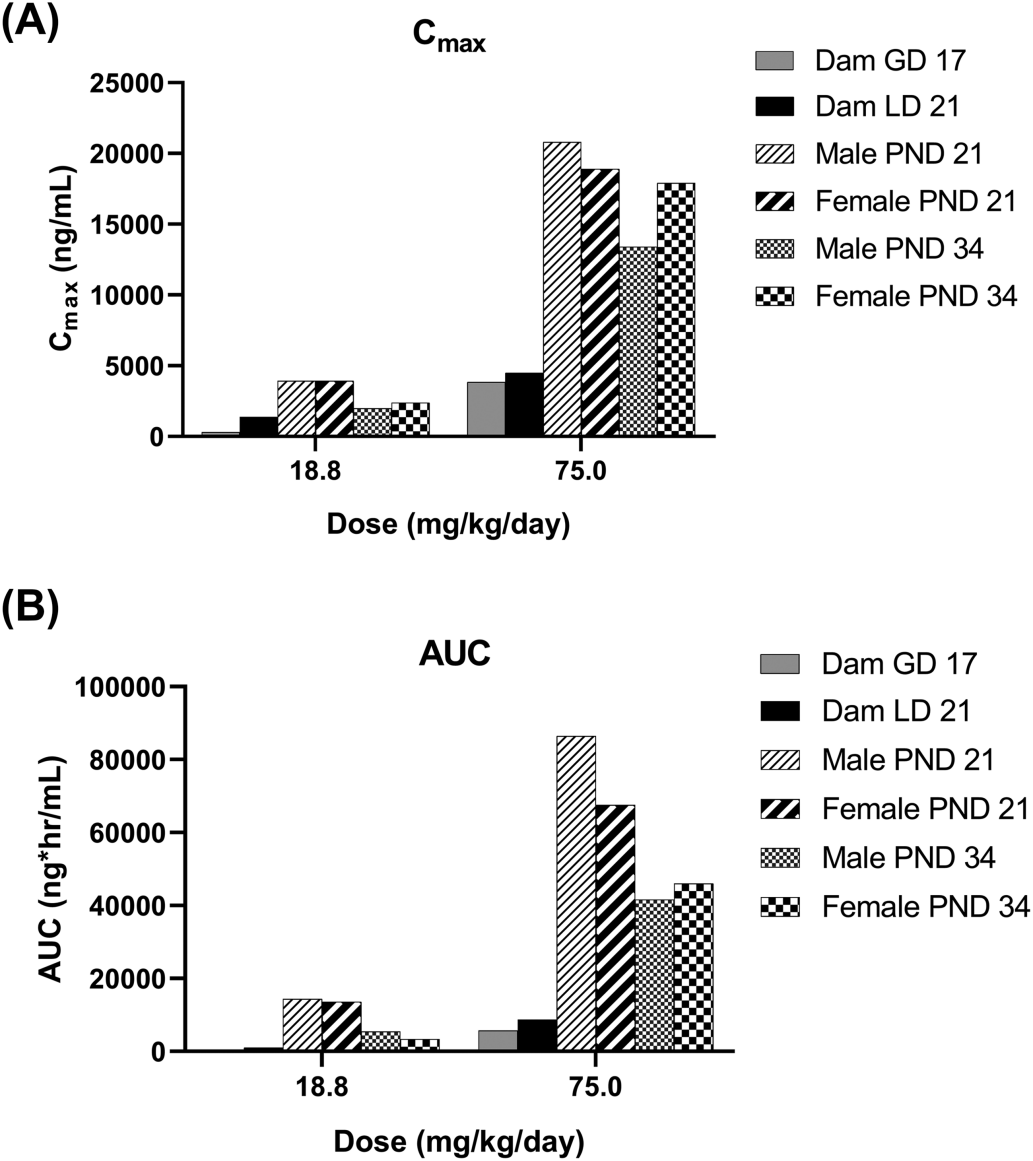
Systemic exposure parameters in rats of varying ages after treatment with hydroxyurea starting on gestational day (GD) 17 in dams and on postnatal day (PND) 10 in offspring. (A) C_max_ and (B) AUC_inf_ estimated in dams on GD 17 and lactation day (LD) 21 and in male and female offspring on PND 21 and 34

In PND 21 pups, the T_max_ of HU in plasma was 0.67 and 0.33 h in the 18.8 and 75 mg/kg/day dose groups, respectively, for both males and females. The dose‐normalized C_max_ and AUC were 1.2‐fold to 1.5‐fold higher, respectively, in the 75 mg/kg/day group compared to the 18.8 mg/kg/day group. Systemic exposure parameters in the pups were higher than those in dams. Specifically, C_max_ was 2.8‐fold to 4.6‐fold higher and AUC was 7.8‐fold to 14.3‐fold higher in pups. Half‐life ranged from 0.7 to 1.5 h. In PND 34 pups, the T_max_ of HU in plasma ranged from 0.33 to 0.67 h, across dose groups and sexes. With increasing dose, C_max_ and AUC increased in a greater‐than‐dose proportional manner, indicated by higher dose‐normalized C_max_ and AUC in the 75 mg/kg/day group compared to the 18.8 mg/kg/day group. The half‐life of HU in PND 34 pups ranged from 1.3 to 2.3 h. There were no apparent differences in TK parameters between the sexes on either PND 21 or PND 34. For comparison, C_max_ and AUC for postnatal timepoints and for dams are plotted in Figure 5.

## 4 DISCUSSION

There is great benefit in using HU to treat children with sickle cell anemia as it improves clinical presentations of the disease, decreases the number of painful vaso‐occlusive crises, reduces the number of transfusions and hospitalizations needed, improves blood flow to organs, and increases survival (Hankins et al., 2008; Kinney et al., 1999; McGann & Ware, 2015; Quinn et al., 2010; Thornburg et al., 2012). However, it is important to improve our understanding of the long‐term health effects of HU treatment, particularly when it is administered starting in childhood. The goal of the studies reported here was to identify doses that could be used to evaluate the potential for adverse effects of HU when administered perinatally and throughout childhood.

In this study, there were no effects of HU on maternal health or littering parameters at the doses administered during late gestation. The decrease in food consumption starting on LD 14 in the 75 and 100 mg/kg/day groups does not necessarily suggest maternal toxicity. Lower food consumption, particularly later in the lactation period, may be due to lower consumption of food by the pups and reflect the offspring toxicity observed, such as decreased body weights and clinical observations. The lack of maternal toxicity at these doses is consistent with the few other oral gavage studies in pregnant rats. No maternal toxicity or only slight reductions in body weight were observed in dams given 200 mg/kg/day HU from GD 7 to GD 20 (Price et al., 1985a, 1985b).

Findings observed in nonclinical pediatric studies can aid in apprising and appreciating the potential contributory response of “developmental age” on the drug's toxicological profile within a species, as well as the identification of novel findings that may inform clinical human use. In this study, rat offspring exposed to HU had lower body weights and exhibited skin discoloration and alopecia at doses ≥75 mg/kg/day. These effects both began around PND 16 in males and females, shortly after they began receiving HU directly. Based on the timing of these observations, it is likely that these effects were due to direct effects of HU on the offspring and not due to gestational exposure. Alopecia is listed as a nonadverse side effect on the HU product sheet (Bristol‐Myers Squibb Company, 2019) and is likely due to effects of HU on highly proliferative cell populations like those in hair follicles. HU (10 mg/kg) administered subcutaneously to newborn SD rats has been demonstrated to interfere with DNA synthesis and mitosis in the epidermis and hair follicles (Stoughton, 1971). The yellow skin discoloration is consistent with jaundice (hyperbilirubinemia) and suggests a severe hemolytic event, or severe liver injury or hepatitis. Indications of liver effects in nonclinical studies are limited to a report of hemosiderosis in the liver or spleen in subchronic rat studies (Bristol‐Myers Squibb Company, 2012). Postmarket reports of cholestasis and hepatitis are listed in HU drug label; liver side effects have not been reported in children (Bristol‐Myers Squibb Company, 2012). Overall, in this study, pregnant rats given ≤150 mg/kg/day showed no signs of toxicity. However, in offspring, postnatal treatment with doses ≥75 kg/kg/day of HU was accompanied by decreases in body weight and adverse clinical observations. The clinical observations of alopecia and skin yellowing observed in the current study are already noted as HU‐induced side effects in adult patients and would likely be expected to be observed in pediatric patients. Nonetheless, the current study apprises the clinical community that these may occur at a lower dose and/or greater severity in this population.

In addition to identifying tolerated doses, we assessed if there was gestational and/or lactational transfer of HU as well as if there were differences in HU toxicokinetics due to age in a limited design. In all studies, HU was rapidly absorbed and eliminated, with plasma concentrations peaking within 1 h after administration and then reaching levels close to the LOD of the assay ˂6 h after dose administration. These kinetic characteristics are similar to what has previously been observed in animals and in humans (NTP‐CERHR, 2008). The half‐life in the present study ranged from 0.5 to 1 h in pregnant dams. Other studies in pregnant rats typically administered HU intraperitoneally and report half‐lives ranging from 15 m to 1.5 h (Philips et al., 1967; Rajewsky, Fabricius, & Hülser, 1971; Wilson, Scott, Ritter, & Fradkin, 1975). In these studies, the half‐life of HU in fetuses was longer than that in dams. In the present study, which administered HU orally, fetuses on GD 17 had a shorter half‐life than dams but higher systemic exposure. Given that HU is given orally to sickle cell individuals, our TK data are a valuable addition to the literature to inform future studies assessing safety for this indication of HU.

There was generally a higher than dose‐proportional increase in C_max_ and AUC with increasing dose, which has also been observed in another study in adult Sprague Dawley rats (Morton et al., 2015) and after intraperitoneal administration of HU in mice (Iyamu, Lian, & Asakura, 2001). In the mouse study, this phenomenon was attributed to a saturation of metabolism because of concomitant decreases in clearance. In both humans and rodents, approximately half of the drug is estimated to be metabolized in the liver through hydrolysis and oxidation reactions; the other half is eliminated unchanged in the urine within the first 24 h (Adamson et al., 1965; Glover, Ivy, Orringer, Maeda, & Mason, 1999; Jiang et al., 1997; Lockamy et al., 2003; Neil Dalton et al., 2005). However, the specific enzyme responsible for the hydrolysis of HU in the liver is still unknown (Colvin & Bono, 1970; Huang et al., 2006). Further characterization of HU metabolism and clearance are needed to explain these findings.

Significant transfer of HU across the placenta was observed on GD 17 in the present study. Similarly, HU crossed the placenta in pregnant rhesus monkeys and Wistar rats injected intraperitoneally with 100 mg/kg HU (Wilson, Scott, Ritter, & Fradkin, 1975). In both species, HU concentrations in the embryo were equal to or higher than maternal plasma concentrations, and elimination was faster in the mother than in the fetus. Findings from our study are similar to the Wilson et al. (1975) study in that fetal HU concentrations were the same or higher than dam plasma HU concentrations. However, in our study, the half‐life of HU was similar between dams and fetuses. There are presently no data available to estimate fetal exposure to HU in humans but given that solute carrier transporters that can transport HU are present in the placenta, such as organic anion transporter 1A2 and organic cation/carnitine transporter 1 (Walker et al., 2017; Walker, Franke, Sparreboom, & Ware, 2011), it is likely that human fetuses also experience some exposure from the mother.

Under the conditions of this study, plasma of PND 14 pups did not have detectable levels of HU, suggesting a lack of transfer of HU from the mother to pups via lactation. HU has been reported to be excreted in human milk, as indicated on the drug label and in a limited number of human case studies (Bristol‐Myers Squibb Company, 2019; Marahatta & Ware, 2017; Sylvester, Lobell, Teresi, Brundage, & Dubowy, 1987). Notably, in a pharmacokinetic study of 16 lactating mothers (14 healthy, 2 with sickle cell anemia), measurable concentrations of HU were found in plasma and breast milk after ingestion of 1 g of HU (Ware et al., 2018). Plasma concentrations in mothers peaked around 60 to 120 m after dosing and ranged from 10 to 40 ng/ml. There was rapid equilibration of HU between milk and plasma as concentrations of HU in breast milk were ~80% of plasma concentrations. Importantly, the profile of HU concentrations in plasma and milk over time was similar between healthy mothers and mothers with sickle cell anemia. There may be species differences between rats and humans in the lactational transfer of HU.

The half‐life of HU was similar between PND 21 and PND 34 animals, and there did not appear to be a sex difference in TK among the offspring at either age. Interestingly, younger animals tended to have higher systemic exposure, indicated by the higher C_max_ and AUC values (Figure 5). For example, AUCs in PND 21 pups were 7.8‐fold to 14.3‐fold higher than that in LD 21 dams and 1.5‐fold to 4.1‐fold higher than that in PND 34 pups. The higher AUC and C_max_ in younger animals could be due the development of clearance or metabolism processes which occurs early in postnatal life. In support of this hypothesis, it is known that elimination of HU occurs primarily through renal organic anion transporters (Walker et al., 2011); expression of this class of transporters increases with postnatal kidney development in rats and potentially in humans (Sweeney et al., 2011).

Based on a limited number of studies on pharmacokinetics of HU in children, the kinetics of HU may change with age in humans. Half‐lives in children range from 1 to 2 h (Estepp et al., 2016; Ware et al., 2011). Because the half‐life of HU in adults has been reported to range from 2 to 5 h, it was suggested that children have faster clearance of HU. However, de Montalembert et al. reported similar half‐lives between children and adults (~6 h) but greater half‐life and AUC in children when compared to infants (de Montalembert et al., 2006). Thus, although the elimination of HU in young rats appears to emulate that observed in children, how HU elimination changes with age may differ between humans and rats. As more data in children become available, this relationship should be reassessed.

Human translatability is a key consideration in animal studies and is typically done by comparing internal dose. The recommended dosing paradigm for HU in children is a starting dose of 20 mg/kg/day followed by dose escalation until mild myelosuppression is obtained and tolerated by the patient (Heeney & Ware, 2008). Using pharmacokinetic data from a clinical study, an AUC of 115 μg * h/ml has been proposed as a target AUC for the initial HU dose in children (Dong, McGann, Mizuno, Ware, & Vinks, 2016). In children given an average of 20 mg/kg daily, the AUC of HU ranged from 68 to 116 μg * h/ml (Estepp et al., 2016; Nazon et al., 2019). In our study, young rats administered 75 mg/kg/day had AUC values of 41–86 μg * h/ml, which approximates the AUC value observed in children. However, considering that we observed offspring toxicity at doses ≥75 mg/kg/day, these data suggest that rats cannot tolerate an HU dosing‐regimen that attains human internal doses. At the lower dose (18.8 mg/kg/day) where there is minimal toxicity, the AUC in rats does not reach even half the recommended AUC in humans. Another study in rats presents data with a similar conclusion. With administration of 500 mg/kg/day of HU orally, adult Sprague Dawley rats had an average AUC of 635 μg * h/ml but also exhibited significant body weight loss, adverse clinical observations, decreased leukocyte counts, and microscopic findings in the thymus, lymph nodes, bone marrow, and gastrointestinal tract (Morton et al., 2015). Exposure multiples of <1 are commonly observed with other small molecule drugs, such as antimitotic cancer chemotherapies, where a similar increase in relative “risk” of an adverse response is acceptable given disease severity and morbidity. Given the TK differences of HU observed between rats and humans, other methods to establish translational relevance of animal studies of HU should be investigated, such as titrating rodent doses based on the pharmacological effect of HU, myelosuppression. This possibility was demonstrated in Lebensburger et al. (2010) where 50 mg/kg/day of HU in SCD mice significantly reduced leukocyte and neutrophil counts and was tolerated by mice for up to 5 months.

## 5 CONCLUSION

HU was administered orally to pregnant Sprague Dawley rats beginning in late gestation and through lactation and to their offspring beginning on PND 10 up to PND 34. Under these conditions, doses ≤150 mg/kg/day of HU were tolerated by dams with no significant effects on littering. However, decreases in body weight and adverse clinical observations were observed in the offspring at doses ≥75 mg/kg/day. TK investigations indicated that HU was transferred to the fetus during gestation but minimally to pups through the milk. The half‐life of HU was not significantly different between sexes. Systemic exposure decreased with increasing age and may be related to the slight increase in the half‐life of HU in younger animals or enzyme ontogeny. Taken together, at doses that were tolerated by rat offspring, the internal dose was much lower than the internal therapeutic level recommended for human children. To overcome these species differences and clarify the potential clinical implications, strategies to translate findings in animals to humans, such as dosing to achieve a similar biological effect, are needed. Overall, these data inform the design of future studies to evaluate the consequences of long‐term HU treatment through childhood. Such studies could include an assessment of the potential for HU to induce reproductive and/or neurological toxicity after early postnatal and juvenile life stage exposure.
